# Guidelines for the establishment and functioning of Animal Ethics Commitees (Institutional Animal Care and Use Committees) in Africa

**DOI:** 10.1177/00236772231178656

**Published:** 2023-09-06

**Authors:** Bert J Mohr, Ouajdi Souilem, Sohair R Fahmy, Francis Fakoya, Khadiga Gaafar, Josiah T Kantiyok, Farida Khammar, Sarrah Mbarek, Lawrence Mugisha, Hany Sleem, Alemayehu Toma, Maricél van Rooyen, Henry Zakumumpa, David I Lewis

**Affiliations:** 1 Scientific Veterinary Consulting, Inc., South Africa; 2 Laboratory of Physiology and Pharmacology, National School of Veterinary Medicine, University of Manouba, Tunisia; 3 Zoology Department, Faculty of Science, Cairo University, Egypt; 4 ACURET.ORG, Nigeria; 5 Johan Vet Network, Nigeria; 6 University of Science and Technology Houari Boumediene, Algiers; 7 Algerian Association of Animal Experimental Sciences, Algeria; 8 High Institute of Applied Biological Sciences of Tunis (ISSBAT), University of Tunis El Manar, Tunisia; 9 College of Veterinary Medicine, Animal Resources and Biosecurity (COVAB), Makerere University, Uganda; 10 National Hepatology and Tropical Research Institute (NHTMRI), Egypt; 11 Egyptian Network of Research Ethics Committees (ENREC), Egypt; 12 Department of Pharmacology, College of Medicine and Health Sciences, Hawassa University, Ethiopia; 13 University of the Free State, South Africa; 14 School of Public Health, Makerere University, Uganda; 15 School of Biomedical Sciences, Faculty of Biological Sciences, University of Leeds, UK; 16 Biological Sciences Teaching Innovation Hub, Faculty of Biological Sciences, University of Leeds, UK; 17 International Union of Basic and Clinical Pharmacology-Integrative and Organ Systems Pharmacology Initiative (IUPHAR IOSP), USA

**Keywords:** 3Rs, ethical review, ethics, ethics and welfare, IACUC or animal ethics committee, policy, quality assurance/control

## Abstract

Animals are used for scientific purposes across Africa to benefit humans, animals or the environment. Nonetheless, ethical and regulatory oversight remains limited in many parts of the continent. To strengthen this governance framework, the Pan-African Network for Laboratory Animal Science and Ethics brought together experts from 12 African countries to create an Africa-centric practical guide to facilitate the establishment and appropriate functioning of Institutional Animal Ethics Committees across Africa. The Guidelines are based on universal principles for the care and use of sentient animals for scientific purposes, with consideration of the cultural, religious, political and socio-economic diversity in Africa. They focus on 11 key elements, including responsibilities of institutions and of the Institutional Official; composition of the Committee; its responsibilities, functioning and authority; ethical application and review processes; oversight and monitoring of animal care and use and of training and competence; quality assurance; and the roles of other responsible parties. The intent is for African institutions to adopt and adapt the guidelines, aligning with existing national legislation and standards where relevant, thus ensuring incorporation into practice. More broadly, the Guidelines form an essential component of the growing discourse in Africa regarding moral considerations of, and appropriate standards for, the care and use of animals for scientific purposes. The increased establishment of appropriately functioning animal ethics committees and robust ethical review procedures across Africa will enhance research quality and culture, strengthen societal awareness of animals as sentient beings, improve animal well-being, bolster standards of animal care and use, and contribute to sustainable socio-economic development.

## Lignes directrices pour la création et le fonctionnement des comités institutionnels de protection et d’utilisation des animaux en Afrique Résumé

Bien que les animaux soient utilisés à des fins scientifiques dans toute l’Afrique pour profiter aux humains, aux animaux ou à l’environnement, le contrôle éthique et réglementaire reste limité dans de nombreuses régions du continent. Pour renforcer ce cadre de gouvernance, le Réseau panafricain pour la science et l’éthique des animaux de laboratoire a réuni des experts issus de 12 pays africains afin de créer un guide pratique centré sur l’Afrique pour faciliter la création et le fonctionnement approprié des comités institutionnels d’éthique des animaux à travers l’Afrique. Les lignes directrices sont fondées sur des principes universels de soin et d’utilisation des animaux sensibles à des fins scientifiques, en tenant compte de la diversité culturelle, religieuse, politique et socio-économique en Afrique. Elles se concentrent sur les 11 éléments clés: Responsabilités des institutions et du fonctionnaire institutionnel; composition du Comité; ses responsabilités, son fonctionnement et son autorité; processus d’application et d’examen éthique; supervision et surveillance des soins et de l’utilisation des animaux, formation et compétences; assurance de la qualité; rôles des autres parties responsables. L’intention est que les institutions africaines adoptent et adaptent ces lignes directrices, en s’alignant sur la législation et les normes nationales existantes, le cas échéant, et en assurant ainsi leur incorporation dans la pratique. Plus largement, elles constituent une composante essentielle du discours croissant en Afrique concernant les considérations morales et les normes appropriées concernant le soin et l’utilisation des animaux à des fins scientifiques. La mise en place accrue de comités d’éthique animale fonctionnant de manière appropriée et de procédures d’examen éthique solides en Afrique permettra d’améliorer la culture et la qualité de la recherche, de renforcer la conscience sociétale des animaux comme êtres sensibles, d’améliorer le bien-être des animaux, de renforcer les normes de soin et d’utilisation des animaux et de contribuer au développement socio-économique durable.

## Leitlinien für die Einrichtung und Arbeitsweise von Tierethikausschüssen (Institutional Animal Care and Use Committees) in Afrika Abstract

In ganz Afrika werden Tiere zu wissenschaftlichen Zwecken zum Nutzen von Menschen, Tieren oder der Umwelt verwendet. Die ethische und gesetzliche Aufsicht ist in vielen Teilen des Kontinents allerdings nach wie vor mangelhaft. Zur Stärkung des Regelungsrahmens hat das Pan-African Network for Laboratory Animal Science and Ethics (Panafrikanisches Netzwerk für Wissenschaft und Ethik von Versuchstieren) Experten aus 12 afrikanischen Ländern versammelt, um einen für Afrika bestimmten praktischen Leitfaden zu erstellen, der die Einrichtung und geeignete Arbeitsweise von institutionellen Ethikausschüssen für Tiere in ganz Afrika unterstützen soll. Die Leitlinien basieren auf universellen Grundsätzen für die Pflege und Verwendung von empfindungsfähigen Tieren zu wissenschaftlichen Zwecken und berücksichtigen die kulturellen, religiösen, politischen und sozioökonomischen Unterschiede in Afrika. Sie konzentrieren sich auf die folgenden 11 Schlüsselelemente: Zuständigkeiten der Einrichtungen und des institutionellen Beauftragten; Zusammensetzung des Ausschusses; seine Zuständigkeiten, Arbeitsweise und Befugnisse; ethische Antrags- und Überprüfungsverfahren; Aufsicht und Überwachung der Pflege und Verwendung von Tieren sowie der Ausbildung und Kompetenz; Qualitätssicherung; die Rolle anderer zuständiger Parteien. Die Leitlinien sollen von afrikanischen Einrichtungen übernommen und angepasst werden, wobei sie gegebenenfalls mit den bestehenden nationalen Rechtsvorschriften und Normen in Einklang zu bringen sind, um so die Anwendung in der Praxis zu gewährleisten. Im weiteren Sinne bilden sie einen wesentlichen Bestandteil des sich in Afrika verstärkenden Diskurses über moralische Erwägungen und angemessene Standards für die Pflege und Verwendung von Tieren zu wissenschaftlichen Zwecken. Die zunehmende Einrichtung angemessen funktionierender Ethikausschüsse für Tiere und solider ethischer Überprüfungsverfahren in ganz Afrika dürfte die Qualität und Kultur der Forschung verbessern, das gesellschaftliche Bewusstsein für Tiere als fühlende Wesen stärken, das Wohlergehen der Tiere verbessern, die Standards für die Pflege und Verwendung von Tieren erhöhen und zu einer nachhaltigen sozioökonomischen Entwicklung beitragen.

## Directrices para la creación y el funcionamiento de comités institucionales para el cuidado y uso de animales en África Resumen

En toda África se utilizan animales con fines científicos en beneficio de las personas, los animales o el medioambiente. No obstante, la supervisión ética y reglamentaria sigue siendo escasa en muchas partes del continente. Para reforzar este marco de gobernanza, la Red Panafricana para la Ciencia y la Ética en el uso de Animales de Laboratorio reunió a expertos de 12 países africanos con el fin de crear una guía práctica centrada en África para facilitar la creación y el funcionamiento adecuado de Comités Institucionales de ética en el uso de animales en todo el continente. Las Directrices se basan en principios universales para el cuidado y uso de animales sensibles con fines científicos, teniendo en cuenta la diversidad cultural, religiosa, política y socioeconómica de África. Se centran en 11 elementos fundamentales: Responsabilidades de las instituciones y de los funcionarios institucionales; composición del Comité; sus responsabilidades, funcionamiento y autoridad; aplicación ética y procesos de revisión; supervisión y control del cuidado y uso de animales, y de la formación y competencia; garantía de calidad; funciones de otras partes responsables. La intención es que las instituciones africanas apliquen y adapten las Directrices, alineándolas con la legislación y las normas nacionales existentes cuando sea pertinente, asegurando así su incorporación a la práctica. A modo más general, las Directrices constituyen un componente esencial del creciente discurso en África sobre las consideraciones morales y las normas apropiadas para el cuidado y el uso de animales con fines científicos. El aumento de la creación de comités de ética en el uso de animales que funcionen adecuadamente y de procedimientos sólidos de revisión ética en toda África mejorará la calidad y la cultura de la investigación, reforzará la conciencia social de los animales como seres sensibles, mejorará el bienestar animal, reforzará las normas de cuidado y uso de los animales y contribuirá al desarrollo socioeconómico sostenible.

## Introduction

The African Union Commission’s ‘Agenda 2063 – the Africa we want’ aspiration envisions ‘a prosperous Africa based on inclusive growth and sustainable development’.^1^ Research animals are seen to play a critical role in this sustainable economic development, particularly in improving human and animal health and well-being (UN Sustainable Development Goal (SDG) 3),^2^ sustainable food and agriculture (SDGs 2 and 12) and economic growth (SDGs 8 and 9).

Whilst some African countries have legislation and systems for the ethical and regulatory oversight of the use of animals for scientific purposes, these remain limited in many parts of the continent.^3^ Recognising this call for change, the African Union’s Animal Welfare Strategy in Africa^4^ articulates a vision of ‘an Africa where animals are treated as sentient beings, as a leading continent in implementation of good animal welfare practices for a competitive and sustainable animal resource sector’, with the goal ‘to transform the animal resources sector through adoption of good animal welfare practices for human well-being, sustainable livelihoods, poverty reduction and economic growth’.

Africa faces challenges in implementing this vision. In addition to legal and regulatory constraints, there are limited resources and expertise to develop human capacity in animal research ethics. There is a need to promote animal welfare better, enhance research quality and culture, and harmonise good practice across the continent through co-operation and collaboration.

The Pan-African Network for Laboratory Animal Sciences and Ethics (PAN-LASE)^5^ was created in 2017 as a forum to develop human resources and capacity for the humane and ethical care and use of research animals across Africa. In addition to providing professional educational opportunities, it resolved to create guidelines for the establishment and functioning of Animal Ethics Committees (AECs), otherwise known as Institutional Animal Care and Use Committees (IACUCs), in Africa.

Whilst several global guidelines are recognised for AECs, Africa is unique regarding its cultural, religious, political and socio-economic diversity. Hence, there was a need to create Guidelines that were Africa-centric, which drew on existing good practice and expertise from across Africa^6–9^ and which reflected the continent’s diversity, resources and balances. While several African countries have guidelines, policies and resources for Human Research Ethics Committees,^10^ the same is often not true for AECs, thus the need for AEC Guidelines.

## Rationale for development of the Guidelines

Humans have a moral obligation and ethical responsibility to promote the well-being of sentient animals and to recognise the intrinsic value of animal life. The humane care and use of animals creates awareness of animal welfare, and individual (personal) and institutional cultures of care.^11^ Africa aspires to be a continent with ‘strong cultural identity, common heritage, values and ethics’.^1^

Ethical review is a critical component of the oversight of the care and use of animals for scientific purposes to ensure humane care and use standards. Ethical review of animal research is critical to ensure that research results are reproducible and that research is conducted with due regard to the well-being and sentience of animals. Appropriate ethical and scientific standards for animal research enhances research quality and therefore global acceptance of the science,^12^^,^^13^ in turn enhancing international collaboration, international rankings of educational institutions, career progression and promotion of scientists, and the mitigation of reputational and legal risk. It also promotes public confidence. Robust ethical review is a requirement for publication in international journals, for the welfare accreditation of animal care and use programmes, and as a requirement of funding bodies.

From a national and regional perspective, appropriate ethical standards encourage the national and African advancement of science and technology, improvements to human and animal health, and well-being and realisation of other UN SDGs.^2^

## Collaborative co-creation of the Guidelines

Fourteen African researchers, veterinarians and animal welfare experts from across Africa, all with experience and expertise in the ethics of animal research (i.e. the authors of this paper), convened at the Ecole Nationale de Médecine Vétérinaire de Sidi Thabet, Tunisia, in 2019 and engaged in collaborative discussions to co-create these Guidelines.

The objective was to create a practical guide which would facilitate the establishment and functioning of AECs (i.e. IACUCs) in Africa as fundamental instruments for the responsible oversight of animal care and use for scientific purposes (including for research, testing and teaching).

The Guidelines are aligned with universal principles for the care and use of animals for research and education,^14^^,^^15^ that animals should only be used when there are no valid non-animal alternatives, with consideration of animal sentience, the application of the principals of humane experimental technique (3Rs),^16^ the five freedoms,^17^ the five domains,^18^ the requirement for robust ethical review (with a harm/benefit analysis), and formal approval before studies may commence.^14^^,^^15^

Their content was informed by existing good practice, legal frameworks, standards and policies relating to the care and use of animals for scientific purposes across Africa,^6–9,19–25^ as well as established international codes and guidelines,^14^^,^^15^ with due regard to the rich cultural, religious, political and socio-economic diversity across Africa.

The draft Guidelines were discussed with an additional 20 delegates from 13 African countries attending the PAN-LASE train-the-trainer workshop for animal research professionals, which was run in parallel at the Ecole Nationale de Médecine Vétérinaire, for debate, comments and modifications.

The final set of democratic Guidelines, reported here, was approved by all 34 delegates.

## Scope of the Guidelines

- The Guidelines are intended to be used in conjunction with applicable national legislation. In cases of inconsistencies, national legislation takes precedence over the Guidelines;
- The Guidelines apply to all live non-human vertebrates (i.e. fish, amphibians, reptiles, birds and mammals) and higher invertebrates (i.e. cephalopods and decapods), of all life stages from half of gestation or being capable of independent feeding (whichever comes first), including but not limited to laboratory animals, farm animals, wildlife, free-living animals, domestic animals, feral animals and genetically altered animals;
- The Guidelines apply to all circumstances involving the care and use of animals for scientific purposes, including for research, education (teaching and training), testing, safety and efficacy studies, the use of animals for diagnostic purposes, production of biological products or substances, field trials, conservation studies, observation, environmental studies and regulatory studies to register a product, including animals that are produced for use in scientific purposes;
- AECs may choose to extend the scope of these Guidelines to include other species (e.g. other invertebrate animals), uses of animals (e.g. non-scientific uses) or other aspects (e.g. the use of animal tissues) to meet their specific situational needs.

## Key elements of the Guidelines

The guidelines describe 11 key elements for implementation by AECs, to ensure the appropriate functioning and standards of the committee (Table 1).

**Table 1.** The 11 key elements (fundamental principles) for implementation by Animal Ethics Committees (AECs) to ensure the appropriate functioning and standards of the Committee.

Key element number	Key element (fundamental principle)	Description
1	Responsibilities of institutions and the Institutional Official	Each institution’s legal, moral and ethical responsibilities
		The requirement for a senior leader of each institution to take overall responsibility for the institution’s animal care and use programme
2	Composition of the Committee	The membership of the committee, including roles and responsibilities
3	Responsibilities of the Committee	Oversight of the institution’s entire animal care and use programme, including animal welfare and care, scientific protocol review, post-approval monitoring, and auditing
4	Functioning of the Committee	The procedural mechanisms by which the committee conducts its business in an appropriate and responsible way
5	Authority of the Committee	The powers delegated to the committee to evaluate, approve or reject, inspect, suspend, or require termination of studies or activities
6	Applications for animal ethics review	The minimum information that must be provided for an application to be reviewed by the AEC
7	The ethical review process	The considerations undertaken by the committee in evaluating applications
8	Oversight and monitoring of animal care and use	The requirement for the AEC to monitor and audit all aspects of the institution’s animal care and use programme, to improve and refine practices based on lessons learnt
9	Oversight and monitoring of training and competence	Ensure that all people involved in the care and use of animals have the appropriate education, training and demonstrated competencies relevant to their role on an ongoing basis; ensure that life-long learning opportunities are available
10	Quality assurance of the Committee’s functioning	Institutional and external mechanisms and assessment procedures by which the institution provides independent quality assurance of the committee’s functioning standards
11	Other responsible parties	The roles and responsibilities of individuals, institutional and external bodies, and regulatory bodies in ensuring the humane care and use of animals, with an appropriate and effective culture of care within the institution

### Institutional responsibilities and the Institutional Official

- Institutional Official: This is the authorised member of senior administration or management of the institution who bears ultimate responsibility and accountability for the institutional animal care and use programme and compliance with animal ethics standards. This person should have the authority and budgetary means to ensure appropriate standards;
- The Institutional Official should provide the required resources to enable the AEC to function effectively and fulfil its responsibilities, including administrative support, office space, training of committee members and so on;
- The Institutional Official should appoint the chairperson of the AEC and the AEC members, in consultation with the chairperson of the committee;
- The institution should ensure the independence of the committee in terms of decision making and functioning, including the authority of the committee and upholding of committee decisions;
- The institution should enable mechanisms for smooth and efficient communication between researchers and the committee, conflict resolution, appeals and adjudication of complaints;
- The institution should ensure that appropriate internal and external quality-assurance assessment of the committee’s functioning is undertaken;
- The institution should ensure compliance with relevant legislation;
- The institution should establish appropriate institutional grievance procedures.

### Composition of the Animal Ethics Committee

- The composition of the committee should ensure competent animal ethics review and oversight;
- The impartiality and independence of members should be appropriately considered, including consideration that at least one member should be independent of the institution;
- The chairperson of the committee should report regularly (usually annually) to the Institutional Official;
- The committee’s composition should have fair representation in terms of gender;
- The membership of the committee should enable the committee to fulfil its functions and meet its responsibilities as outlined in these guidelines;
- Membership of the committee should consist of the following:
- at least one veterinarian with the relevant expertise and knowledge;
- at least one scientist with relevant experience in animal research and use;
- at least one public member to represent general community interests, who is independent of the science and care of the animals in the institution and who is not involved in the care or use of animals for scientific purposes;
- Additional members may be included as committee members in order to meet the needs of the committee (e.g. animal care staff, people with knowledge of animal welfare, statisticians, ethicists, people with knowledge of biosafety, people with legal training, etc.). Additional people may be co-opted as required.
- The overall membership of the committee, including additional members, should be representative of the institutional environment, considering the needs of relevant stakeholders. An institutional analysis of animal care and use, including identification of relevant parties, can be beneficial to identify key stakeholders.

### Responsibilities of the Animal Ethics Committee

- The AEC should have oversight of the entire institutional animal care and use programme, including animal acquisition, breeding, transport, husbandry and care, animal welfare assessment, restraint, clinical procedures, killing and safe disposal;
- Protocol definition – a detailed written description of the proposed use of animals for scientific purposes, as submitted to an AEC, in application for review and consideration for approval;
- Ethical review of all protocols involving the care and use of animals for scientific purposes;
- Post-approval monitoring of approved protocols to ensure compliance with approval conditions;
- Inspection of animal facilities and areas to advise on changes needed to improve or ensure animal welfare.
- Report all needs and deficiencies in the animal care and use programme to the Institutional Official.
- Ensure that appropriate mechanisms are created for reporting and investigation of concerns regarding animal welfare or non-compliance;
- Ensure appropriate veterinary care of all animals;
- Keep records of the use of animals for scientific purposes in the institution;
- Provide a forum of discussion to promote a culture of care in the institution;
- Ensure that all AEC members are adequately trained and competent, with reference to international guidelines for animal ethics training;
- Ensure that systems are established to confirm that all relevant animal care and use personnel are adequately trained and competent (see section ‘Oversight of Training and Competence’);
- Report on a regular (usually annual) basis to the institution, including the composition of the committee, meetings held and attendance, challenges faced by the committee, details of protocols reviewed, details of animals bred and used including the severity of studies, unanticipated problems and adverse events encountered (including actions taken), details of non-compliance or misconduct, protocols withdrawn or suspended, challenges encountered in the animal care and use programme with recommendations for improvements, details of facility inspections and other relevant aspects identified by the committee.

### Functioning of the Animal Ethics Committee

- AECs should establish formal documentation and standard operating procedures to describe all relevant aspects of their functioning, including decision-making processes;
- In the interest of transparency, relevant committee documentation should be publicly available;
- The AEC should set timelines for submission and review of protocols, which should be communicated to applicants;
- Adequate records should be kept of AEC activities, including attendance registers and minutes of meetings;
- Committee members should sign an appropriate confidentiality agreement;
- Conflicts of interest of AEC members should be appropriately declared and managed;
- The AEC should establish mechanisms for complaints and concerns relating to the care and use of animals to be raised and investigated.

### Authority of the Animal Ethics Committee

- Evaluate and approve protocols and amendments to protocols (when all relevant requirements are met) for animal care and use;
- Monitor the care and use of animals in approved protocols to establish compliance with approval conditions (i.e. post-approval monitoring of active studies);
- Inspect any facilities or areas where animals are kept or used, including breeding and experimental establishments for compliance on animal welfare aspects;
- Investigate suspected non-compliance or misconduct, or refer investigation of non-compliance or misconduct to the Institutional Official or to a regulatory body, as may be appropriate;
- Suspend or withdraw approval for previously approved protocols in cases of established non-compliance;
- Instruct that an animal(s) is euthanised or receives appropriate treatment in cases of welfare concerns;
- It is essential that the authority of the committee and that of the chairperson, as well as any additional authorities delegated by the Institutional Official, be clearly specified in the formal documentation of the committee, which should be approved by the Institutional Official.

### Applications for animal ethics review

- Applications for the use of animals for scientific purposes should be submitted and approved by the AEC before any of the animals are used for the specified purposes;
- Applications for animal use should be by completion of the relevant AEC protocol application form;
- The minimum details to be included in the protocol application form should enable the AEC to conduct the ethical review as outlined in the section ‘The Ethical Review Process’;
- Applications should provide the rationale for animal use in lay language (i.e. non-specialised terms) so that it is understandable by people who are not subject experts;
- The Principal Investigator (PI; i.e. the person who takes primary responsibility for compliance with the approved conditions of the protocol) should be clearly specified.

### The ethical review process

- The ethical review conducted by the AEC should include consideration of the following: appropriate justification for the use of animals where there are no relevant alternatives; appropriate application of the Three Rs (i.e. Replacement, Reduction and Refinement of the use of animals); a clear valid scientific question building on previous knowledge; clear description of benefits arising; sound experimental design, including estimation of sample size; relevant experience and expertise of involved personnel; training and competence of involved personnel in all animal care and use procedures; species-appropriate animal care and husbandry; suitable species-specific environmental enrichment which enables the animals to exercise as appropriate; a clear description of the procedures performed; a clear description of severity and cumulative harms to the animals (i.e. detrimental impacts on animal well-being, including fear, discomfort, pain, suffering, distress or lasting harm); the clinical progression of the animal’s condition over time; welfare monitoring (i.e. frequency, signs to be monitored, the people responsible); humane end points (i.e. the maximum level of suffering that will be permitted) and the actions to be taken when these are reached; methods for euthanasia and confirming death; appropriate disposal of biological materials; the availability of appropriate facilities; and acceptance by the PI of full primary responsibility for the protocol;
- The AEC should perform a harm/benefit assessment in order to ensure that the likely benefits of the study will outweigh the total cumulative lifetime harms to the animals;^26^
- The AEC may use a checklist for reviewers to harmonise the ethical review process;
- The AEC should ensure that appropriate scientific review of all protocols, including the pedagogical justification where animals are being used for education and training, is conducted, as well as appropriate review of biosafety and occupational health and safety.

### Oversight and monitoring of animal care and use

- Post-approval monitoring of approved protocols to ensure compliance with the conditions of the approval, including the competence of personnel in procedures performed on animals;
- Inspection of facilities or areas where animals are kept and used;
- Progress reports from PIs (annual and final) – issues identified can help improve institutional practice;
- Review and approval of standard operating procedures for animal care and use.

### Oversight of training and competence

- All AEC members should be appropriately trained in relevant animal ethics review processes;
- At least three levels of training should be provided for AEC members: introductory training for new members, in-depth training and continuing professional development;
- Training should include the ethical evaluation process, regulations and guidelines, the 3Rs (Replacement, Refinement and Reduction), animal welfare, humane end points, the harms-benefit assessment, the culture of care, animal facility inspection, the standard operating procedures of the AEC, knowledge and understanding of the key research areas undertaken in the institution, training in committee functioning skills, and relevant biosafety and occupational health and safety training;
- All people who encounter animals used for scientific purposes, or who have an impact on the welfare of these animals, should receive appropriate education and training in applicable scientific and animal welfare aspects relevant to their role, with reference to global guidelines;
- Relevant introductory and continued education training should be provided to people who perform procedures on animals (including researchers, students and staff), people who care for animals (including breeders and suppliers of animals that are used for scientific purposes), people who euthanise animals, and people who design projects and procedures;
- The practical competence of people who perform procedures on animals, euthanise animals or care for animals should be assessed and confirmed by another person who is known to be competent in the relevant techniques and who is trained in assessing competency. Standards for competence may be as determined by a national body or competent authority, or by reference to international guidelines for good practice. Systems should be established to ensure that all people remain competent;
- Veterinary and para-veterinary professionals should receive education and training relevant to their role, including knowledge of the applicable species, systems and procedures.

### Quality assurance of the Animal Ethics Committee’s functioning

- Oversight of the AEC’s functioning is an essential quality-assurance mechanism;
- The chairperson of the AEC should report regularly (usually annually) to the Institutional Official;
- Internal oversight should include institutional assessment of AEC functioning. This can increase the efficiency and performance of the AEC and may contribute to recognition of the AEC and its authority within the institution;
- External (to the institution) oversight may include auditing and/or registration of AECs by non-institutional bodies to confirm appropriate composition and functioning of the committees. This may include national and/or regional oversight mechanisms and may include the compilation of national statistics of animal use for scientific purposes. National Competent Authorities may also play a role in terms of external oversight of facility standards and competence in procedures.

### Other responsible parties

- Veterinarians: In addition to membership of the AEC, the role of veterinarians should include evaluation of animal health and welfare (including preventative care, health monitoring, diagnosis, treatment and post-mortem examination), sharing and promoting activities or approaches which enhance both physiological and psychological aspects of animal welfare, training and evaluation of competence of personnel in animal procedures, evaluation of standards of animal facilities and consultation during the design of protocols. The veterinarian should liaise with the AEC chairperson and the institutional official;
- Researchers, teachers, students, staff and other people with responsibility for the humane care and use of animals for scientific purposes;
- Institutional bodies with oversight of biosafety and occupational health and safety;
- Institutional bodies responsible for the scientific review of proposals for animal use;
- National Competent Authorities may regulate standards of facilities and competence;
- National bodies may regulate standards of animal ethics review and oversight.

## Going forward

These Guidelines provide the basis for the establishment of AECs and of associated robust ethical review implementation at institutions across Africa. We have attempted to reduce the Guidelines to minimum requirements, which should be practically implementable, to encourage institutions to adhere to good practice when establishing AECs. The intent is for African institutions and national councils to adopt and adapt the Guidelines, in line with existing national legislation or standards,^19–25^ other relevant legislation (e.g. for medicines development, wildlife, etc.), taking into account local cultural and religious diversity, and the ownership of animals, to ensure incorporation into practice, that is, the establishment of appropriately functioning institutional AECs that formally review and approve all proposals for animal use before studies are initiated. On a broader level, the Guidelines are intended to encourage the growing discourse in Africa regarding the moral principles and standards for the care and use of animals. The Guidelines provide guidance for institutions seeking to strengthen their existing procedures, harmonised with universal good practice and the African context. It is an opportunity to harmonise practice across the continent for the benefit of all.

The Guidelines may be modified and expanded as AECs are introduced across Africa, and the animal care and use community learn from their experiences. There is a need for ongoing discussions and engagement with stakeholders, including scientific organisations, regulators and institutions across Africa, to encourage the awareness, uptake and implementation of the Guidelines. The Pan-African Network for Laboratory Animal Science and Ethics (PAN-LASE),^5^ in collaboration with key local, national and regional stakeholders, aims to drive this vision forward.

The purpose or aim of developing these Guidelines was to empower and support Institutions and individuals in establishing and operationalising AECs across Africa. The Guidelines are very much the first step. Going forward, as AECs become more commonplace and embedded across the continent, these Guidelines can be revised and refined, incorporating additional elements or good practice from elsewhere in the world. This could include broadening the recommended membership of the committee; the requirement that all those involved in the care and use of research animals have an education and understanding of the 3Rs; consideration of who (e.g. veterinarian, AEC chair, etc.) should have 24/7 access to research animals; inclusion and guidance on undertaking harm-benefit analyses; and recommendations for reporting of progress to Competent Authorities. These suggestions, and others, could be a starting point for the working party creating version 2 to focus on.

The establishment of more AECs and robust ethical review systems across Africa will strengthen the societal awareness of animals as sentient beings, improve animal well-being, strengthen the humane care and use of animals for scientific purposes, enhance research quality and culture, and contribute to sustainable socio-economic development.
